# Characterization of Na^+^ and Ca^2+^ Channels in Zebrafish Dorsal Root Ganglion Neurons

**DOI:** 10.1371/journal.pone.0042602

**Published:** 2012-08-03

**Authors:** Yu-Jin Won, Fumihito Ono, Stephen R. Ikeda

**Affiliations:** 1 Section on Transmitter Signaling, Laboratory of Molecular Physiology, National Institute on Alcohol Abuse and Alcoholism, National Institutes of Health, Bethesda, Maryland, United States of America; 2 Section on Model Synaptic Systems, Laboratory of Molecular Physiology, National Institute on Alcohol Abuse and Alcoholism, National Institutes of Health, Bethesda, Maryland, United States of America; University of Waterloo, Canada

## Abstract

**Background:**

Dorsal root ganglia (DRG) somata from rodents have provided an excellent model system to study ion channel properties and modulation using electrophysiological investigation. As in other vertebrates, zebrafish (*Danio rerio*) DRG are organized segmentally and possess peripheral axons that bifurcate into each body segment. However, the electrical properties of zebrafish DRG sensory neurons, as compared with their mammalian counterparts, are relatively unexplored because a preparation suitable for electrophysiological studies has not been available.

**Methodology/Principal Findings:**

We show enzymatically dissociated DRG neurons from juvenile zebrafish expressing *Isl2b*-promoter driven EGFP were easily identified with fluorescence microscopy and amenable to conventional whole-cell patch-clamp studies. Two kinetically distinct TTX-sensitive Na^+^ currents (rapidly- and slowly-inactivating) were discovered. Rapidly-inactivating *I_Na_* were preferentially expressed in relatively large neurons, while slowly-inactivating *I_Na_* was more prevalent in smaller DRG neurons. RT-PCR analysis suggests *zscn1aa*/*ab*, *zscn8aa/ab*, *zscn4ab* and *zscn5Laa* are possible candidates for these *I_Na_* components. Voltage-gated Ca^2+^ currents (*I_Ca_*) were primarily (87%) comprised of a high-voltage activated component arising from ω-conotoxin GVIA-sensitive Ca_V_2.2 (N-type) Ca^2+^ channels. A few DRG neurons (8%) displayed a miniscule low-voltage-activated component. *I_Ca_* in zebrafish DRG neurons were modulated by neurotransmitters via either voltage-dependent or -independent G-protein signaling pathway with large cell-to-cell response variability.

**Conclusions/Significance:**

Our present results indicate that, as in higher vertebrates, zebrafish DRG neurons are heterogeneous being composed of functionally distinct subpopulations that may correlate with different sensory modalities. These findings provide the first comparison of zebrafish and rodent DRG neuron electrical properties and thus provide a basis for future studies.

## Introduction

Dorsal root ganglia (DRG) are the metamerically organized peripheral sensory ganglia involved in transmitting somatosensory information from the body to the central nervous system. DRG contains heterogeneous subpopulations of neurons based on neuroanatomical, immunohistochemical and electrophysiological criteria. Different somal diameters are well-correlated with the conduction velocity of attached axons and distinct sensory modalities. For example, rapidly conducting Aα/Aβ-type neurons have the largest cell bodies and transmit proprioceptive and tactile information. Conversely, slower conducting Aδ- and C-type DRG neurons have smaller cell bodies and are involved in nociception [1]–[4]. In rodents, isolated DRG neuron somata have provided an excellent model system for electrophysiological investigation. Thus, numerous studies have characterized rodent DRG subpopulations in regard to ion channel expression, biophysical properties, and modulation [5]–[8].

As in other vertebrates, zebrafish (*Danio rerio*) primary sensory neurons are found within DRG and possess peripheral axons that bifurcate into dorsal and ventral territories in each body segment allowing sensory information to flow from the periphery to the CNS. Anamniote vertebrates also possess a unique transient peripheral sensory neural population termed Rohon-Beard (R-B) neurons. R-B neurons appear early in development and are subsequently replaced, functionally, by DRG neurons. Following the disappearance of R-B neurons, DRG neurons, together with the lateral line system, represent the primary means of detecting mechanical stimuli [9]. To date, anatomical and developmental studies of zebrafish DRG neurons have primarily focused on embryonic periods [10]–[12]. As zebrafish mature, the number of DRG neurons increases, the optical clarity decreases, and anatomical accessibility is reduced. Unlike in rodents, a preparation of DRG neurons from juvenile/adult zebrafish suitable for electrophysiological studies has not been available. Thus, the biophysical characteristics of ion channels in adult fish DRG neurons are relatively unexplored.

Here, we show that enzymatically isolated zebrafish DRG neurons are readily identified and suitable for whole-cell voltage-clamp studies using an *Isl2b*-promoter driven EGFP transgenic zebrafish line, *Tg(Isl2b:EGFP)^ZC7^*, that facilitates identification of sensory neurons. Using this technique, we characterized functionally expressed voltage-activated Na^+^ and Ca^2+^ channels from juvenile zebrafish DRG neurons. Our results indicate that, as in higher vertebrates, zebrafish DRG neurons are heterogeneous being composed of functionally distinct subpopulations that may correlate with different zebrafish sensory modalities. These findings provide the first comparison of zebrafish and rodent DRG neuron electrical properties and thus provide a basis for future studies of the evolutionary origin of pain sensation and modulation of neuronal excitability.

## Results

### Preparation of single DRG neurons from *Tg(isl2b:EGFP)^ZC7^* zebrafish

**Figure 1:**
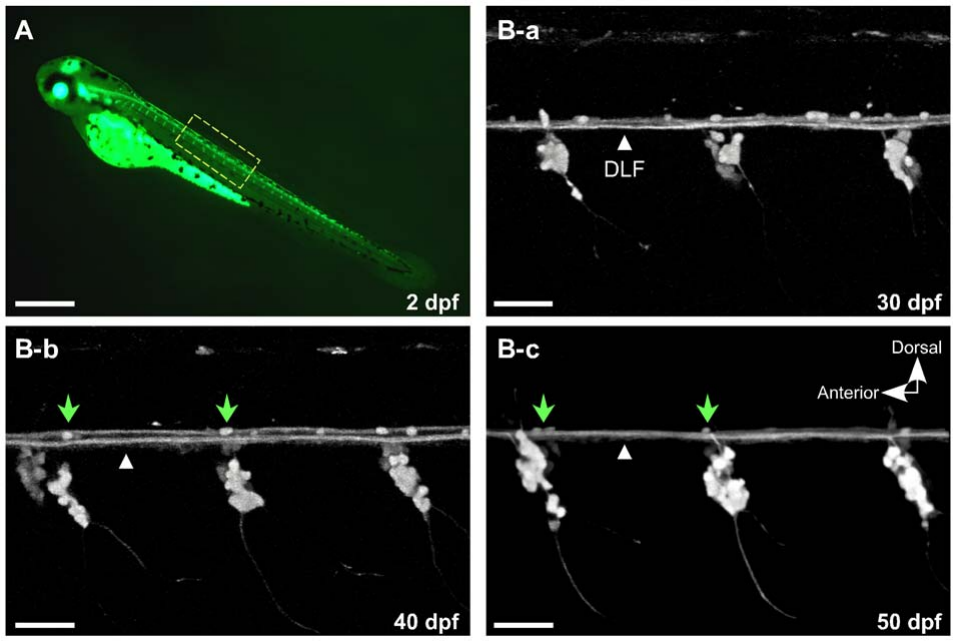
EGFP expression driven by the *Isl2b* promoter facilitates observation of dorsal root ganglia development concurrent with degeneration of Rohon-Beard (R-B) neurons. ***A***, At 2 days post-fertilization (dpf), EGFP was intensely expressed in R-B and DRG neurons. ***B-a–c***, Higher magnification confocal images of the spinal cord in *isl2b:EGFP* at 30, 40 and 50 dpf. A few EGFP-labeled cells remained over the dorsal longitudinal fasciculus (DFL, white triangles) that progressively decreased until only ∼1–3 cells/field remained (B-b and c, green arrow heads). All images are maximum intensity projections of z-stacks acquired in the lateral plane using confocal microscopy. The contrast of the images was adjusted to emphasize EGFP-labeled sensory neurons. Scale bar in A represents 0.5 mm and the other scale bars represent 50 µm.

To obtain identified DRG neurons, a transgenic zebrafish line *isl2b:EGFP* was selected [13] (Fig. 1A). In a previous study, the development of peripheral sensory neurons was followed in *isl2b:EGFP* zebrafish embryos [14] and R-B neurons were observed for up to two weeks post fertilization. Figure 1B-a–c depict sensory neurons expressing EGFP, driven by the *isl2b* promoter, in the trunk of a living *isl2b:EGFP* embryo imaged using confocal microscopy at 30, 40 and 50 day post-fertilization (dpf), respectively. DRG are present bilaterally in every trunk segment. At these observation periods, additional scattered EGFP fluorescence was present dorsal to the dorsal longitudinal fasciculus (DLF). It is not clear whether these fluorescent puncta represents remnants of degenerating R-B neurons or other cell types labeled by the *isl2b* promoter. Assuming the former, one would predict functional impairment based on the shrunken cell bodies and absence of axonal trajectories. At 50 dpf, the dorsal fluorescence has mostly disappeared with the exception of ∼1–3 puncta which remain in the vicinity of each DRG (Fig. 1B-c, arrow heads). To minimize contamination from these non-DRG cell types, we utilized *isl2b:EGFP* juvenile fish around 50 dpf for the DRG neuron preparation.

**Figure 2:**
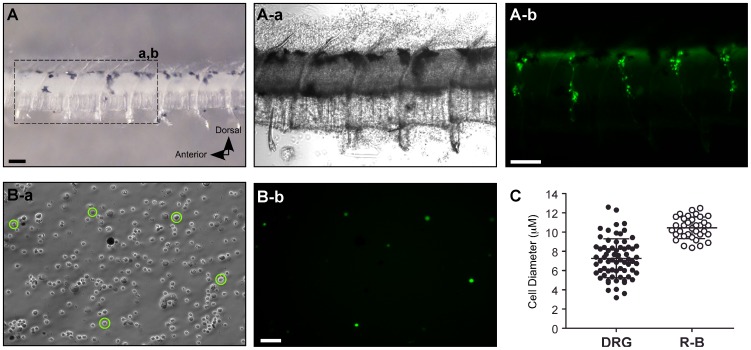
Isolation of single DRG neurons from *isl2b:EGFP* transgenic zebrafish. ***A***, Dissected central trunk region including the spinal cord and notochord from juvenile *isl2b:EGFP* fish (left panel). Enlarged dashed region shows EGFP-labeled DRG present in every trunk segment from the primary preparation (A-a and b). Note that the trunk segment had been exposed to enzymes at this point. ***B***, Phase-contrast (B-a) and fluorescent (B-b) photomicrographs of acutely dissociated DRG neurons (circled) from the primary preparation. Scale bar in A (top) represent 0.1 mm and scale bar in B (bottom) represent 50 µm. ***C***, Dot plots representing cell diameter of single dissociated DRG (filled circles) and R-B (open circles) neurons. Error bars represent standard deviation and statistical significant was determined using an unpaired t-test with Welch's correction.

The cranial portion of the fish containing additional sensory neurons expressing EGFP under the control of *isl2b* promoter was removed as in the previous study [14]. To increase enzyme penetration as well as to avoid contamination from other EGFP expressing cells, the central trunk region including the intact spinal cord and notochord was dissected (Fig. 2A). DRGs were present in every trunk segment and could be identified by EGFP fluorescence from this primary preparation (Fig. 2A-a and b). Consequently, many different cell populations including EGFP-labeled DRG neurons were obtained following enzymatic and mechanical dissociation (Fig. 2B). The acutely dissociated individual DRG neurons were spherical and devoid of neuronal processes— thus facilitating spatial clamp control during whole-cell patch-clamp studies.

**Figure 3:**
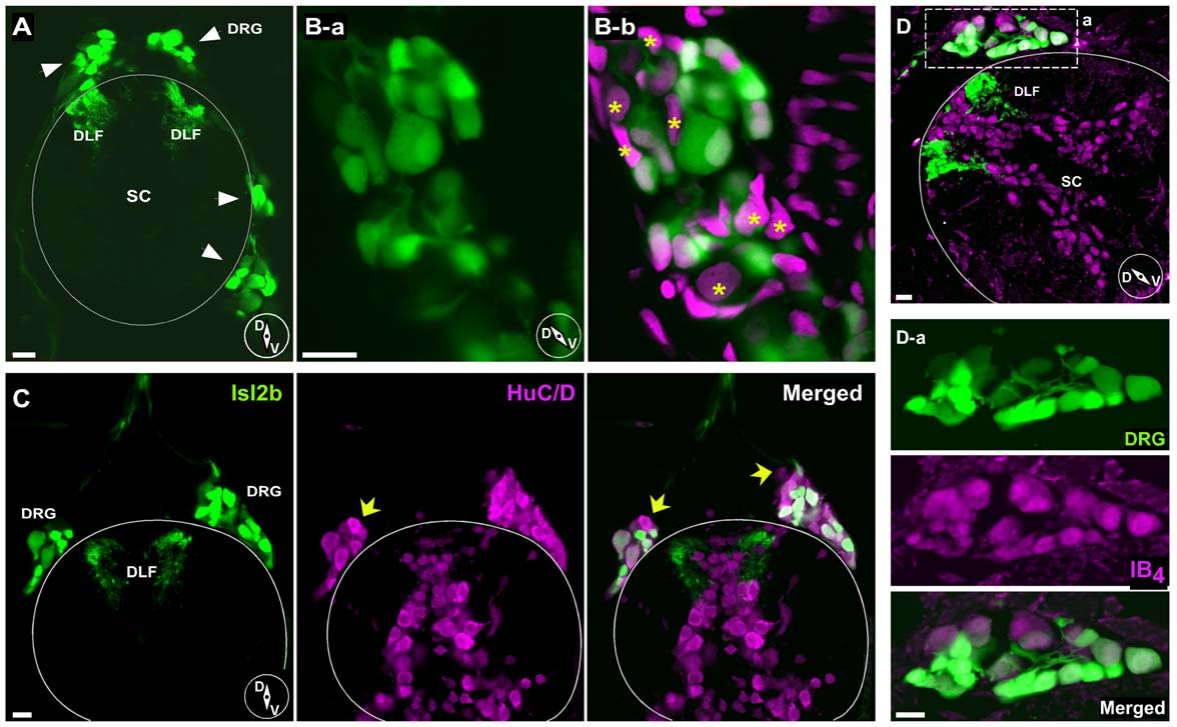
Immunohistochemistry of DRG neurons in juvenile *isl2b:EGFP* fish sections. ***A***, Confocal image displaying EGFP-labeled DRG neurons and DFL in transverse section. Note that both dorsal and ventrolateral positioned DRG (arrows) cell bodies were observed. ***B***, Higher magnification images of DRG ganglion reveal a variety of cell body sizes (B-a). Counterstaining with DAPI (B-b, magenta) revealed that some EGFP-negative or dim cell bodies were also present in DRG sections (asterisks). ***C***, EGFP-positive and -negative DRG neurons from juvenile *isl2b:EGFP* fish. A few DRG neurons stained with anti-HuC/HuD neuronal protein antibody (magenta) did not express EGFP. ***D***, Isolectin B_4_ (IB_4_) staining. Most DRG cell bodies and some spinal neurons were labeled with IB_4_ (magenta). Dashed rectangular region was enlarged for displaying labeled DRG neurons (D-a). Solid line represents the dorsal boundary of the spinal cord. Inset cartoons represent orientation of images. D, dorsal; V, ventral; SC, spinal cord; DFL, dorsal longitudinal fasciculus. All scale bars represent 10 µm.

There was a large variance in the diameter of dissociated single DRG neurons when compared with the relatively homogeneous R-B neuronal population (latter data from Won et al., 2011). R-B neurons were grouped around a mean diameter of 10.5 µm (range 8.4–12.5 µm; n = 34), while DRG neurons varied over a much wider range (range 3.2–12.6 µm; n = 64) with a mean of 7.3 µm (Fig. 2C, *p*<0.05, Unpaired t-test with Welch's correction). To examine heterogeneity of DRG soma sizes *in situ*, frozen transverse sections of *isl2b:EGFP* fish were prepared. Confocal imaging of the sections revealed that each DRG ganglion contained a variety of cell body sizes and was located adjacent to the spinal cord bilaterally (Fig. 3A). Occasionally, some cell bodies were observed either more dorsolaterally or dorsomedially adjacent to the notochord as previously reported [10]. Counterstaining with DAPI revealed that some EGFP-negative cell bodies were present in the DRG sections (Fig. 3B, stars). To address what fraction of DRG neurons in *isl2b:EGFP* juvenile fish express EGFP, frozen sections were stained with an antibody, Mab 16A11, that recognizes a subset of vertebrate ELAV-related RNA binding proteins HuC/HuD expressed specifically in neurons [15]. As shown in Figure 3C, a considerable number of DRG neurons labeled with Mab 16A11 did not express overt EGFP. This result implies that a subset of, but not all, DRG neurons from juvenile fish express *isl2b*.

In an attempt to correlate EGFP expression with biochemical phenotype, immunohistochemistry with some common mammalian sensory neuron markers was performed. Most EGFP-expressing DRG neurons were labeled with isolectin B_4_ (IB_4_) from *Griffonia simplicifolia*. However, there were IB_4_-labeled neurons that were devoid of detectable EGFP expression. In mammalian systems, IB_4_-positive neurons are believed to be nociceptive non-peptidergic neurons which respond to glial cell line-derived brain-derived neurotrophic factor [16], [17]. We also tested the monocloncal antibody clone N52, which recognizes the high-molecular weight (200 kD) neurofilament protein that characterizes sensory neuron somata that give rise to myelinated axons (A-fibers). However, N52 antibodies did not appear to stain zebrafish DRG neurons despite displaying robust staining of mammalian DRG neurons (data not shown). These results indicate that well-established markers for mammalian primary sensory neurons subtypes may not function identically in zebrafish sensory neurons due to nonconserved epitopes or other factors.

### Voltage-gated *I_Na_* in DRG neurons

**Figure 4:**
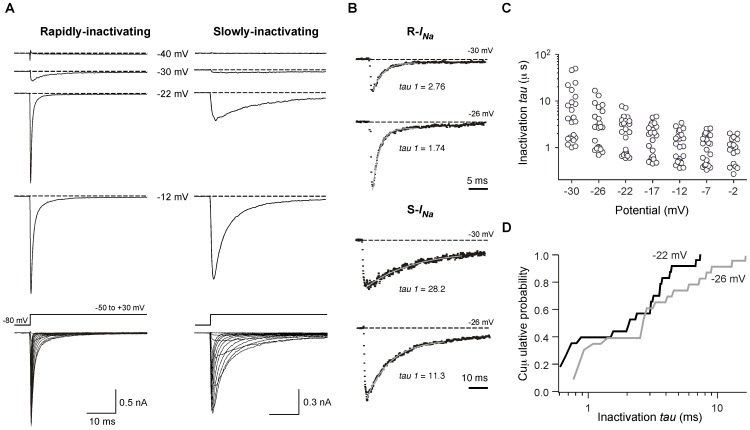
Distinct subpopulations of DRG neurons based on Na^+^ current (*I_Na_*) inactivation kinetics. ***A***, Representative traces ensemble for Rapidly/Slowly-inactivating *I_Na_* acquired using whole-cell patch-clamp. Holding potential: −80 mV; test potentials are indicated for each trace. Superimposed Rapidly/Slowly-inactivating *I_Na_* were evoked by a series of voltages steps to potentials ranging between −70 and +40 mV from a holding potential of −80 mV (bottom). For illustrative purposes, current traces from −50 to +30 mV are shown. ***B***, Curve fitting examples and time constants (*tau*) for the decay phases from Rapidly/Slowly-inactivating *I_Na_* traces. Single exponential functions are represented by gray lines. Holding potential: −80 mV; test potentials are indicated for each trace. ***C***, Dot plots represent current decay/inactivation time constant fitted to a single exponential process at the indicated potential. ***D***, Cumulative probability distribution of inactivation *ταυ* values obtained at the indicated conditioning potentials.

**Figure 5:**
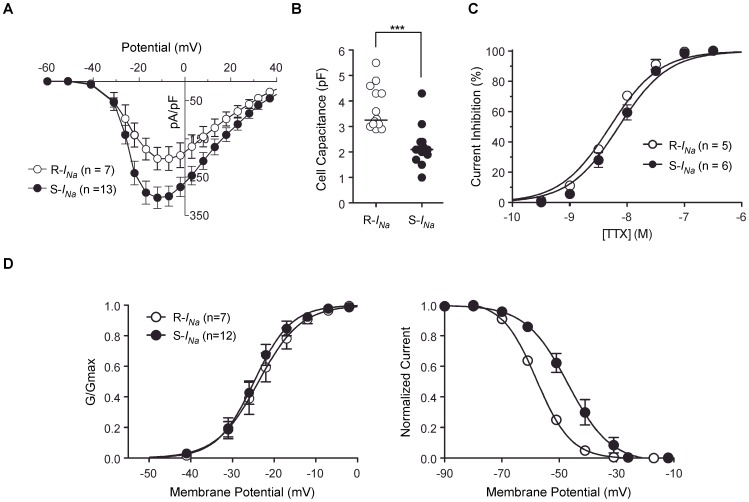
Characterization of R-and S-*I_Na_* recorded in zebrafish DRG neurons. ***A***, The current density versus test potential (*I–V*) relationship for R- and S-*I_Na_*. ***B***, Dot plots represent the membrane capacitance of DRG neurons displaying R- (open circles) and S-*I_Na_* (filled circles). Vertical bars represent the median. *** P<0.001, Mann-Whitney *U*-test. ***C***, Concentration-response curves for block of the R- and S-*I_Na_* by tetrodotoxin (TTX). Solid lines represent nonlinear regression least-square fits of experimental points to a Hill equation (see under *Material and Methods*). ***D***, The voltage-dependence of the Na^+^ conductance (*G_Na_*, left panel) and steady-state inactivation (right panel) of R- and S-*I_Na_*. Conductance was calculated from *G_Na_ = I_Na_/(V_m_−V_rev_)*, in which *I_Na_* is the peak current, *V_m_* is the potential of the test pulse, and *V_rev_* is the reversal potential for *I_Na_*. The solid line represents a nonlinear regression fit to the Boltzmann function: 1/(1+exp[−(*V*−*V_1/2_*)/*k*]), where *V* is the step membrane potential, *V_1/2_* is the half-activation potential, and *k* is a slope factor. Voltage-dependence of inactivation was determined using a 200 ms conditioning pulse followed by a test pulse to −10 mV. Test pulse currents were normalized to the maximal value. Solid line is a fit to the Boltzmann equation (*k* is negative for inactivation curve). Error bars on each symbol represent the mean ± s.e.m. The number of neurons tested is shown in parentheses.

Voltage-gated Na^+^ currents (*I_Na_*) from EGFP-expressing single dissociated juvenile zebrafish DRG neurons were recorded using the whole-cell variant of the patch-clamp technique in solutions designed to isolate *I_Na_* (see Materials & Methods). The currents were elicited by command pulses from holding potential of −80 mV to the potentials indicated. *I_Na_* recorded from individual neurons were either rapidly- or slowly-inactivating currents based on inactivation kinetics (Fig. 4A). All EGFP-expressing neurons examined expressed *I_Na_*. The majority of *I_Na_* current decays were well fit to a single exponential process and most attempts to fit a double exponential function to the current decay did not significantly improve the fitting parameters (Fig. 4B). However, some DRG neurons (27%) that displayed rapidly inactivating current decay were better fit to a double exponential process and thus co-expression of Na^+^ channel types in a minority of neurons is possible. In contrast, the slowly inactivating current decays were well described by a single exponential process plus non-inactivating component (14%). The single time constant for individual neurons was plotted at the indicated potential (Fig. 4C) to functionally segregate the population with the caveat that this represents a simplification of a potentially complex phenomenon. The time course of current activation was insufficiently resolved for detailed kinetic analysis. Inactivation time constants at −30 mV ranged from about 1 ms to greater than 30 ms with visual evidence for a multimodal distribution in the scatter plot at most voltages (Fig. 4C). To examine the distribution further, the cumulative probability of inactivation *tau* values were plotted at the indicated potentials. The plot for inactivation at −22 mV, especially, displayed a distinct flattening around 1 ms (Fig. 4D) suggesting that at least two populations of DRG neurons expressing functionally distinct Na^+^ channels were present. Based on this analysis, DRG neurons were classified into two groups: those expressing rapidly-inactivating-*I_Na_* (R-*I_Na_*), comprising about 40% of the neurons, and slowly-inactivating-*I_Na_* (S-*I_Na_*). Figure 5A depicts the current density versus test potential (*I–V*) relationship for the two types *I_Na_* in DRG neurons. The *I–V* relationships were superficially similar; the currents began to activate around −40 mV, reached a peak near −12 mV, and declined approaching an extrapolated reversal potential of about +45 mV. Interestingly, R-*I_Na_* were present in relatively large DRG neurons with whole-cell capacitances ranging between 2.9 and 5.5 pF with a median of 3.3 pF. Conversely, S-*I_Na_* arose from smaller DRG neurons with a median of 2.1 pF (range 1.0–4.3 pF, *p*<0.001, Fig. 5B). DRG neurons in rodents display a number of slowly activating and inactivating *I_Na_* components that are resistant to the blocker tetrodotoxin (TTX) [18]. However, both type of *I_Na_* in zebrafish DRG neurons were completely abolished by 100 nM TTX, a concentration that spares mammalian TTX-resistant currents (IC_50_ for R-*I_Na_* = 5.0±0.03 nM; S-*I_Na_* = 7.1±0.04 nM; Fig. 5C). To examine voltage-dependent properties of activation, the normalized conductance was calculated from the peak of R-and S-*I_Na_* current traces using the chord conductance equation and plotted versus the test potential (Fig. 5D, left). The smooth curves are fits to a Boltzmann function with midpoints (V_50_) of −23.8±0.6 mV and a slope of 5.1±0.6 mV (slope factor, *k*) for R-*I_Na_* and −24.8±0.4 mV and 4.4±0.4 mV for S-*I_Na_*. Steady-state inactivation was determined using a voltage protocol comprised of a 200 ms conditioning pulse to voltages between −90 mV and −10 mV (Fig. 5D, right) followed by a constant test pulse to −10 mV. In contrast to V_50_ of activation, the half-inactivation potential for S-*I_Na_* shifted by ∼10 mV in the depolarizing direction (R-*I_Na_* = −57.9±0.4 mV; S-*I_Na_* = −46.9±1.0 mV). The slope factor, *k*, for R-*I_Na_* (6.0±0.3 mV) and S-*I_Na_* (7.3±1.0 mV) did not differ significantly.

### Na^+^ channel isoforms in DRG neurons

**Figure 6:**
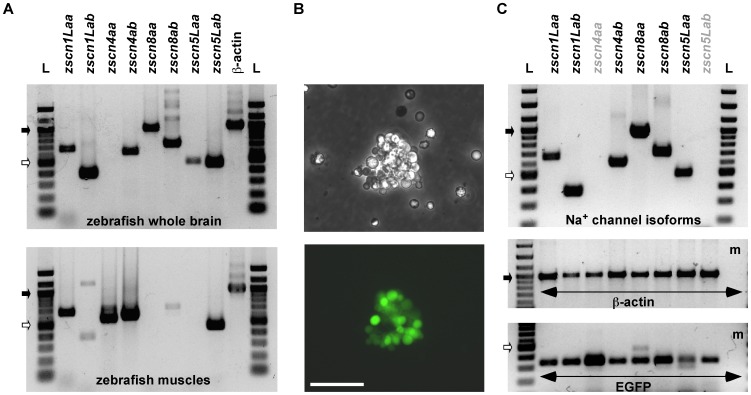
RT-PCR analysis of mRNA encoding Na^+^ channels from zebrafish DRG. ***A***, Upper and lower electrophoresis images show RT-PCR products generated from specific primer sets coding for Na^+^ channel isoforms (*zscn1Laa/ab*, *zscn4aa/ab*, *zscn8aa/ab* and *zscn5Laa/ab*) from zebrafish whole brain and muscles, respectively. ***B***, Phase-contrast (upper) and fluorescent (bottom) images of an example dissociated a DRG cluster as was used for RT-PCR. Scale bar represent 50 µm. ***C***, RT-PCR products generated from a DRG cluster. PCR products of each lane represent they were generated from same RT reaction tube. *Zscn* primer sets were used in the same order as arranged in Fig. 6A. PCR was also performed with β-actin as well as EGFP primer sets to establish successful DRG neurons isolation. The resultant PCR products were separated and visualized on 1.5% agarose gel. L: DNA ladder, m: culture media, Filled and opened arrows represent 1 Kbp and 0.5 Kbp size markers, respectively.

The R-*I_Na_* of DRG neurons exhibited biophysical properties similar to those reported for *I_Na_* of R-B mechasnosensory neurons [14], [19]. However, *I_Na_* with slower inactivating components have not been reported for R-B sensory neurons. As the presence of discrete kinetic components suggested the presence of several Na^+^ channel isotypes in zebrafish DRG neurons, we examined expression of Na^+^ channel mRNA using RT-PCR analysis. As a first step, we performed RT-PCR using gene duplicated Na^+^ channel isoforms specific primer sets (*zscn1Laa/ab*, *zscn4aa/ab*, *zscn8aa/ab* and *zscn5Laa/ab*) on total mRNA isolated from zebrafish whole brain and muscles. Gel electrophoresis revealed bands compatible with the predicted product size of the primer sets (659/380, 573/605, 964/707 and 479/461 bp, respectively). Most of Na^+^ channel isoforms transcripts were detected in zebrafish whole brain sample with the exception of *zscn4aa*. In contrast, skeletal muscle displayed a more restricted expression profile with *zscnL1aa*, *zscn4aa/ab*, and *zscn5Lab* producing the most prominent bands (Fig. 6A). We then attempted RT-PCR analysis with single dissociated zebrafish DRG neurons. Neither Na^+^ channels nor β-actin transcripts were successfully amplified from single neurons (data not shown). Next, we developed a preparation comprised of several DRG neurons to increase the template and hence quantity of RT-PCR product. Clusters of EGFP expressing DRG somata were separated from the spinal cord preparation (Fig. 6B). As an internal reference, β-actin mRNA was amplified and an EGFP primer set was used to establish successful EGFP labeled neurons isolation (Fig. 6 C, middle and bottom panel). Of the tested Na^+^ channel isoform primer sets, *zscnL1aa/ab*, *zscn4ab*, *zscn8aa/ab* and *zscn5Laa* produced products of the appropriate size (Fig. 6C, top panel). Nomenclature of *SCNA* gene and their expression patterns are summarized in Table 1. This result may account for the presence of S-*I_Na_* in DRG neurons encoded from non-neuronal types of Na^+^ channel isoforms such as *zscn4ab* or *zscn5Laa*.

**Table 1:** Nomenclature of Scna gene and mRNA expression patterns in zebrafish.

**Mammalian gene**	*SCN1A*	*SCN2A*	*SCN3A*	*SCN9A*	*SCN4A*	*SCN8A*	*SCN5A*	*SCN10A*	*SCN11A*
**Protein**	Na_v_1.1	Na_v_1.2	Na_v_1.3	Na_v_1.7	Na_v_1.4	Na_v_1.6	Na_v_1.5	Na_v_1.8	Na_v_1.9
**Rodent DRG**	**+**	**+**	**(+)** a	**+**	**−**	**+**	**+**	**+**	**+**
***D. rerio*** ** orthologues**	*zscn1Laa/ab*				*zscn4aa/ab*	*zscn8aa/ab*	*zscn5Laa/ab*		
***D. rerio*** ** DRG**	**+/+**				**−/+**	**+/+**	**+/−**		
***D. rerio*** ** Brain**	***+/+***				**−/+**	**+/+**	**+/+**		
***D. rerio*** ** Muscle**	***+/−***				**+/+**	**−/−**	**−/+**		

a , embryonic distribution; +, detected; −, not detected.

### Voltage-gated *I_Ca_* in DRG neurons

**Figure 7:**
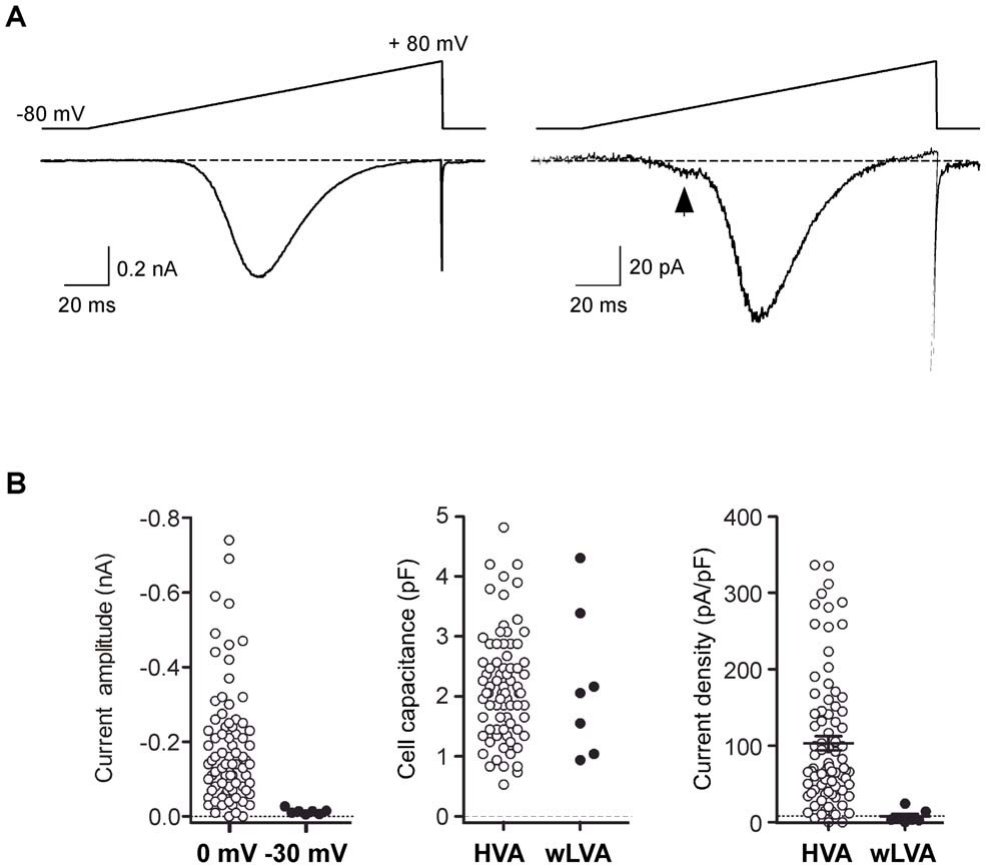
Ca^2+^ currents (*I_Ca_*) from single dissociated DRG neurons. ***A***, Two representative traces of *I_Ca_* acquired from separated neurons using whole-cell patch-clamp. *I_Ca_* were evoked by a 160 ms ramp from −80 to +80 mV (top of traces) from a holding potential of −80 mV. A tiny “hump” (arrow) in the ramp current, indicative of low-voltage-activated *I_Ca_* was observed in some neurons (right). ***B***, Dot plots representing current amplitude (left panel), membrane capacitances (middle panel) and current density (pA/pF, right panel) of DRG neurons with (closed circles) and without (filled circles) low-voltage-activated components. Statistical significance was determined using an unpaired t-test with Welch's correction for unequal variance (P>0.05, middle panel). Error bars represent s.e.m.

**Figure 8:**
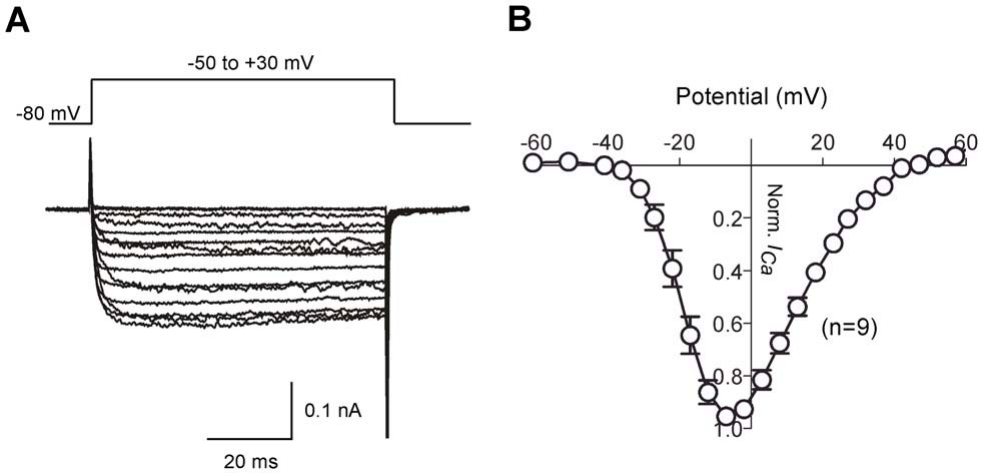
Ca^2+^ current-voltage (*I*–*V*) relationships from zebrafish DRG neurons. ***A***–***B***, Superimposed *I_Ca_* traces (A) and means normalized (to maximal amplitude) *I*–*V* relationships (B). Current traces were evoked by a series of voltages steps to potentials ranging between −80 to +60 mV from a holding potential of −80 mV. For illustrative purposes, only traces from −50 mV to −30 mV are shown. Error bars on each symbol represent the mean ± s.e.m. The number of neurons tested is shown in parentheses.

Ca^+^ currents (*I_Ca_*) from single dissociated DRG neurons were recorded using the whole-cell variant of the patch-clamp technique in solutions designed to isolate *I_Ca_* (see Materials & Methods). Voltage-activated inward *I_Ca_* was evoked by a 160 ms ramp command pulse from holding potential of −80 to +80 mV (Fig. 7A). Most neurons displayed a high-threshold inward *I_Ca_* component, based on the ramp *I–V* curve, which initiated near −35 mV and displayed a monotonic activation component (Fig. 7A, left). A few neurons displayed a small but prominent hump in the *I*–*V* curve at hyperpolarized potentials (−60 to −30 mV) in addition to a high-threshold current component (Fig. 7A, right). DRG neurons displaying both low-voltage activated- (LVA−) and high-voltage activated- (HVA−) *I_Ca_* comprised 8% (0.03–0.16, 95% CI, modified Wald method) of the total recorded cell population. The mean *I_Ca_* amplitude for LVA- and HVA- components was −10±3 (n = 7) and −235±30 pA (n = 84), respectively (Fig. 7B, left panel). The membrane capacitance (C_m_) of neurons with and without LVA- component was not significantly different (P>0.05, unpaired t-test with Welch's correction, Fig. 7B, middle panel). Mean current densities, determined with 10 mM Ca^2+^ as the charge carrier, were 103.2±9 and 8.3±2 pA/pF for HVA- and LVA-*I_Ca_* components, respectively (Fig. 7B, right panel). As the number of DRG neurons possessing both *I_Ca_* components was relatively low (8%) and the amplitude of LVA-*I_Ca_* was small, further characterization of *I_Ca_* focused on DRG neurons displaying only HVA-*I_Ca_*. Figure 8 depicts normalized (to maximal currents) *I_Ca_*-*V* relationships and superimposed current traces evoked by depolarizing pulses (−50 to +30 mV) from a holding potential of −80 mV. *I_Ca_* began to activated around −40 mV, reached a peak near 0 mV, and declined thereafter. The *I_Ca_* trajectory showed little inactivation during a 60 ms test pulse (Fig. 8, left panel).

### Pharmacology of HVA-*I_Ca_* in DRG neurons

**Figure 9:**
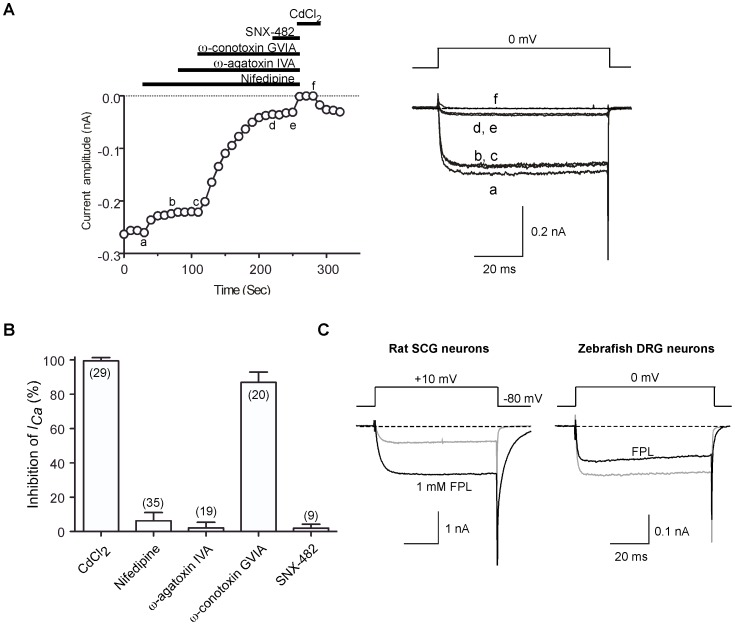
Pharmacological dissection of HVA-*I_Ca_* in zebrafish DRG neurons. ***A***, *Left*, Time courses of *I_Ca_* amplitude during serial application of nifedipine (10 µM), ω-agatoxin IVA (0.5 µM), ω-conotoxin GVIA (3 µM), SNX-482 (300 nM) and CdCl_2_ (100 µM). *I_Ca_* was evoked every 10 s by 70 ms test pulses to 0 mV from a holding potential of −80 mV. The horizontal bars indicate the duration of drug application. *Right*, superimposed current traces obtained at different time points during drug application (labeled as a–f). ***B***, Bar graph representing the mean *I_Ca_* inhibition (%) produced by application of the indicated antagonists or toxins. Error bars represent s.e.m. The number of neurons tested is indicated in parentheses. ***C***, Effect of non-dihydropyridine Ca^2+^ channel agonist FPL 64176 (FPL) on *I_Ca_* in DRG neurons. FPL (1 µM) was applied to rat superior cervical ganglion (SCG) neurons as a positive control (left panel). Note that FPL applied to zebrafish DRG *I_Ca_* display neither an increase in macroscopic inward currents nor greatly prolonged trajectory of the tail currents (right panel).

We next sought to identify the types of HVA-*I_Ca_* channels functionally expressed in DRG neurons by applying antagonists and toxins that occlude specific Ca^2+^ channel types [20], [21]. Figure 9A illustrates the time course of *I_Ca_* amplitude reduction following sequential cumulative application of different subtype-specific Ca^2+^ channel blockers. Application of 10 µm nifedipine [22] a blocker of the L-type (Ca_V_1.x) channel family, produced a slight inhibition (8.1%) of *I_Ca_*. In the continued presence of nifedipine, application of ω-agatoxin IVA (0.5 µm), a P/Q-type (Ca_V_2.1) channel blocker, produced no additional overt reduction of *I_Ca_*. Addition of ω-conotoxin GVIA, an N-type (Ca_V_2.2) channel blocker [23], [24], to this mixture at a saturating concentration (3 µm) resulted in an 80% reduction of total *I_Ca_*. Lastly, SNX-482 (300 nM), a selective antagonist of recombinant α1E (Ca_V_2.3) channels [25] was applied to DRG neurons but failed to block the residual *I_Ca_*. The small residual current remaining following application of the four antagonists was abolished by application of the non-selective Ca^2+^ channel blocker CdCl_2_ (100 µm). As shown in Figure 9B, 100±0.3% (n = 29) of total inward current was the CdCl_2_-sensitive *I_Ca_*, and 87±1.4% (n = 20) of total *I_Ca_* were blocked by ω-conotoxin GVIA. We conclude from these data that N-type Ca^2+^ channels (Ca_v_2.2) underlie the vast majority of VGCCs in DRG neurons, and other Ca^2+^ channel isoforms (L-, P/Q- and SNX-482 sensitive R-type) contributed the remaining 11% of the total *I_Ca_*. Lastly, to provide further evidence for the presence of L-type Ca^2+^ channels in DRG neurons, we applied FPL 64176 (FPL), a non-dihydropyridine Ca^2+^ channel “agonist” that increases macroscopic inward current through L-type Ca^2+^ channels and slows deactivation [26]. As a positive control, we applied FPL (1 µm), to isolated rat superior cervical ganglion (SCG) neurons. Under these conditions, peak inward *I_Ca_* increased and deactivation was retarded as evident from the prolonged trajectory of the tail currents (Fig. 9C, left). Conversely, FPL applied to zebrafish DRG *I_Ca_* decreased peak current amplitude (17±3%, n = 7) and produced only a modest slowing of tail currents (Fig. 9C, right). As FPL is thought to be specific for L-type Ca^2+^ channels, these results suggest that a minor component of *I_Ca_* might arise for L-type channels in zebrafish DRG neurons. However, it appears that the pharmacological effects of FPL on zebrafish Ca^2+^ channels are not completely comparable to those documented for mammalian preparations thus confounding this interpretation.

### G-protein modulation of *I_Ca_* in DRG neurons

**Figure 10:**
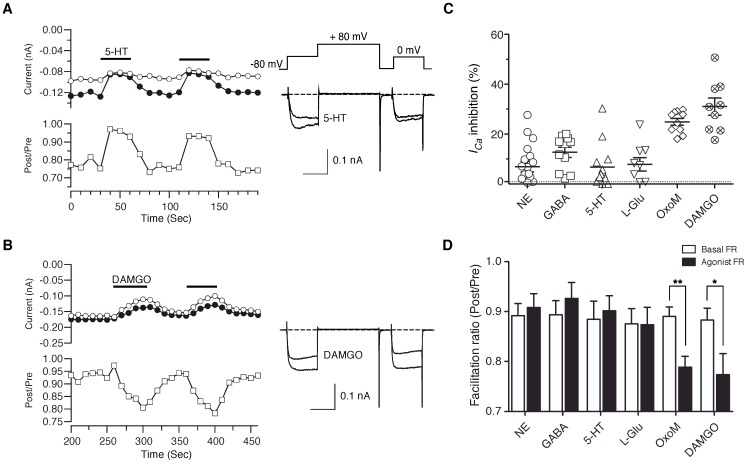
Modulation of HVA-*I_Ca_* by neurotransmitters in zebrafish DRG neurons. ***A–B***, Time courses of *I_Ca_* (left) and superimposed current traces (right) evoked with the double-pulse voltage protocol (inset on the traces in A) during application of 5-HT (10 µM) and DAMGO (1 µM), respectively. The *I_Ca_* amplitude generated by the pre-pulse (filled circles) and post-pulse (open circles) are plotted. Facilitation ratio (FR) was calculated as the ratio of the post-pulse to pre-pulse *I_Ca_* amplitude (open squares). The horizontal bars indicate the duration of drugs application. ***C***, Dot plots represent inhibition of prepulse *I_Ca_* (%) produced by application of norepinephrine (NE, 10 µM), GABA (100 µM), 5-HT, l-glutamic acid hydrochloride (l-Glu, 100 µM), oxotremorine M (OxoM, 10 µM) and DAMGO. ***D***, Summary of the mean FR (Post/Pre) before (basal, open bars) or after (filled bars) application of agonists. Data are presented as mean ± sem. * *P*<0.05, ** P<0.001 by paired *t*-test.

To examine the G-protein modulation of Ca^2+^ channels in DRG neurons, *I_Ca_* was evoked by a modified double-pulse voltage protocol [27] consisting of a test pulse to 0 mV, a strong depolarizing conditioning pulse to +80 mV (prepulse), and second identical test pulse (postpulse) to 0 mV (Fig. 10A, right panel inset). A parameter known as the facilitation ratio (FR) is determined from the ratio of the postpulse to prepulse current amplitude and is often used as metric for voltage-dependent Ca^2+^ channel inhibition mediated by Gβγ subunits [28], [29]. Thus, an increase in FR during drug application, termed voltage-dependent inhibition, is suggestive of a Gβγ-mediated mechanism whereas little change or a decrease in FR, voltage-independent inhibition, implies a different signaling pathway (e.g., Gα_q_-mediated inhibition). In the absence of G-protein coupled receptor (GPCR) agonists, the basal FR is an indicator of tonic G-protein activation [30]. The mean basal FR of DRG neurons was 0.91±0.01 (n = 60), which indicates little tonic G-protein activation. A basal FR≪1, seen in some neurons (e.g. Fig. 10A), is indicative of voltage-dependent inactivation.

To identify natively expressed GPCRs that modulate Ca^2+^ channels in DRG neurons, we screened a panel of agonists that activate adrenergic (norepinephrine, 10 µm), GABAb (GABA, 100 µm), serotonin (5-HT, 10 µm), metabotropic glutamate (l-glutamate, 100 µm), muscarinic acetylcholine (oxotremorine-M, 10 µm) or μ-opioid (DAMGO, 1 µm) receptors. Figure 10A and B illustrate representative time courses of prepulse (closed circles), postpulse amplitude (open circles) and FR (open squares) prior to, during (black line), and after agonist washout. Extracellular perfusion of 5-HT resulted in *I_Ca_* inhibition via a voltage-dependent mechanism (Fig. 10A). The onset and washout of agonist effect was rapid occurring within one or two 10 s test pulses. The *I_Ca_* modulation displayed the hallmarks of voltage-dependent Gβγ-mediated inhibition, namely slowing of activation during the prepulse, relief of inhibition during the postpulse, reversal of kinetic slowing in the postpulse, and increased FR [29], [30]. Similar phenomenology was seen following application of NE, GABA and l-glutamate but the responses were not present in every cell (Fig. 10C). Given the limited sample sizes, it was unclear whether these variations in response correlated with somal diameter. In contrast, application of either DAMGO or OxoM produced a voltage-independent inhibition of *I_Ca_* based on the absence of overt kinetic slowing in the prepulse trace or increase in the FR during agonist-mediated inhibition (Fig. 10B). Mean *I_Ca_* inhibition by DAMGO and OxoM were 25±1 and 31±3%, respectively and these responses were relatively consistent when compared with other tested neurotransmitters; inhibition was >15% in all tested neurons and the coefficient of variation (CV) was 17.6 and 34%, respectively. Conversely, voltage-dependent modulations by NE, GABA, 5-HT and l-glutamate displayed a variable response with a CV of 54–133% (Fig. 10C). Change of FRs during agonist application are shown in Fig. 10D. Voltage-dependent modulation by NE, GABA, 5-HT and l-glutamate produced increase of FRs. Conversely, application of either DAMGO or OxoM resulted in a decrease of FR.

**Figure 11:**
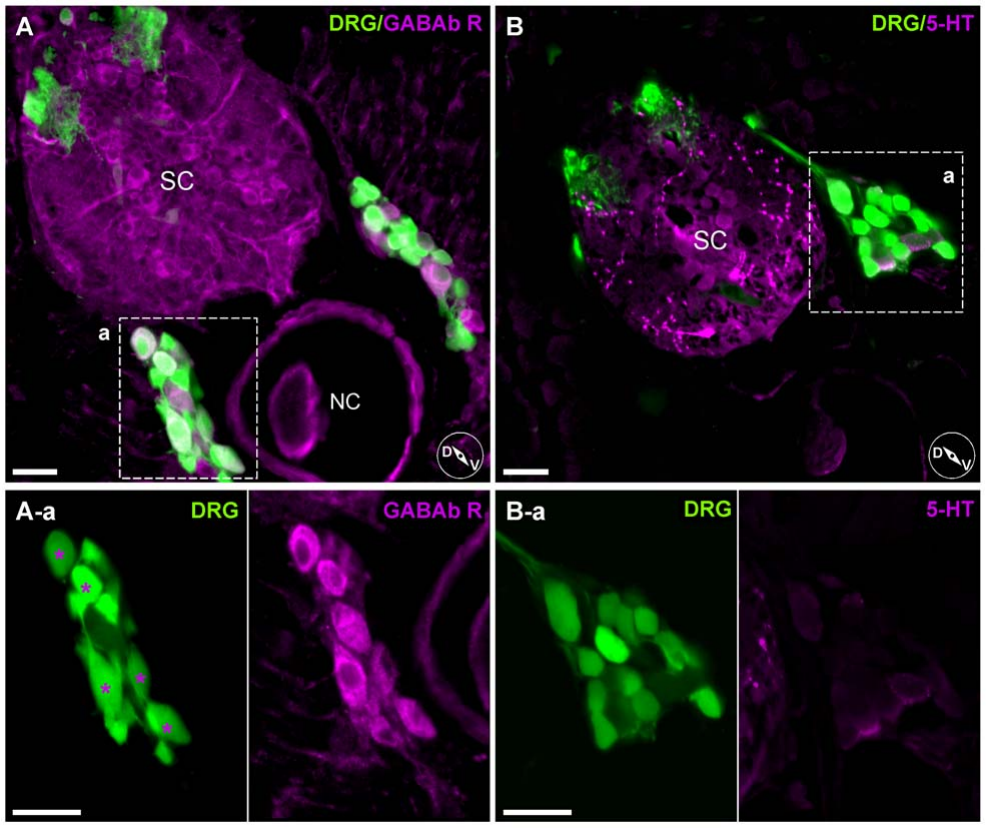
Heterogeneous expression pattern of anti-GABAb receptor antibody staining on DRG neurons. ***A***, Labeling with an anti-GABAb R1 antibody. Enlarged images (A-a, left and right) show only a specific subset of DRG neurons were labeled (asterisks). ***B***, Labeling with an antibody against 5-HT. 5-HT antibody binding was mostly in spinal cord tracts rather than DRG neurons somata (enlarged box B-a). Inset cartoons represent orientation of images. D, dorsal; V, ventral; SC, Spinal Cord; NC, Notochord. Scale bars represent 20 µm.

To assess whether variability in *I_Ca_* modulation arose from variability in GPCR expression, immunohistochemistry was performed for GABAb R (Fig. 11A). Photomicrographs of transverse sections of *isl2b:EGFP* juvenile fish show a specific subset of DRG neurons that were labeled with the GABAbR antibody (Fig. 11A-a, asterisks). As a negative control, immunostaining with an antibody against 5-HT was performed. As previously reported [31], 5-HT antibody binding was mostly in spinal cord tracts rather than DRG neurons somata (Fig. 11B). These results suggested that variable responses of *I_Ca_* by neurotransmitters in DRG neurons likely arise from differential expression patterns of GPCR.

## Discussion

The objectives of the present study were threefold. First, to develop a preparation of isolated zebrafish DRG neurons suitable for whole-cell patch-clamp investigation. Second, to characterize the neurons in terms of morphology, physiology, and pharmacology. We were particularly interested in the biophysical properties of the voltage-gated Na^+^ and Ca^2+^ currents and the modulation of the latter channels by GPCR stimulation. Third, we wished to determine if *Danio rerio* sensory neurons were advantageous for investigating molecular mechanisms of ion channel modulation. Zebrafish, as a model vertebrate organism, possess several desirable traits such as economy of rearing, optical clarity during early stages of development, and ease of producing transgenic progeny. However, whether the properties of zebrafish DRG neurons were comparable to their mammalian counterparts was unknown.

### Preparation of DRG neurons from juvenile *isl2b:EGFP* zebrafish line

We took several precautions when isolating DRG neurons from other EGFP-expressing neurons present in the *isl2b:EGFP* line. First, the cranial region containing EGFP-labeled neurons was removed prior to dissection. Second, the central trunk region, including the intact spinal cord and notochord, was isolated by dissection to minimize contamination from potential EGFP-labeled cells arising from ventral portions of the trunk. Since most EGFP-labeled R-B somas disappear during larval stages [9], [32], the dorsal trunk portion of juvenile fish was relatively free of *EGFP*-labeled non-DRG neurons. Third, we considered migratory DRG neurons which leave the DRG and acquire a sympathetic neuron phenotype [33], [34]. Although potentially present in our preparation, these neurons are rare under basal conditions (e.g., 2 neurons per embryo at 4 dpf) and would thus comprise a small fraction of the total isolated cell population.

As the DRG neurons were isolated from juvenile *isl2b:EGFP* fish, we wondered whether the entire DRG population was labeled. Based on immunostaining and confocal imaging (Fig. 3), we concluded that a subset, estimated to be the majority but not quantified, of DRG neurons express EGFP at levels readily detectable with fluorescence (both widefield and confocal) microscopy. In vertebrates, the *isl-1* gene family is expressed in specific subsets of neurons and has been closely associated with the determination of neuronal fates [35]. In zebrafish embryos, *isl2b* mRNA is initially expressed ubiquitously but gradually becomes restricted to specific tissue subsets including eyes and the tectal region of the mesencephalon [36]. Thus, *isl2b* expression in DRG neurons may be initially widespread but becomes progressively more restricted as development proceeds. As EGFP fluorescence was used to identify the neurons, the properties reported here necessarily reflect the characteristics of this subgroup. Whether the unlabeled neuron population differs in these properties remains unclear.

### Morphological characteristics of zebrafish DRG and isolated neurons

Unlike mammalian DRG, zebrafish DRG are dynamic into the early adult stage both increasing in neuron numbers [10] and shifting macroscopically in position. Initially, the DRG appear near the junction of the ventral spinal chord and dorsal notochord. During later development (e.g., 30–50 dpf), the ganglia elongate with the cellular mass extending dorsally (Fig. 1). Some sections show labeled neurons occupying a space dorsal to the spinal chord (Fig. 3). This dorsal location was unexpected as previous studies, typically performed in less mature fish, have illustrated DRG exclusively in a ventral location. However, an early study of zebrafish neuroanatomy [37] described spinal ganglia in mature zebrafish as “typically found dorsolateral to the cord, wedged in between it and the muscle mass”. This location is consistent with our observations in some sections (e.g., Fig. 3). It is likely that the laterally located R-B neurons identified by Weis (1968) in 18 dpf fish were actually dispersed DRG neurons similar to those illustrated in Figure 3A.

Comparison of isolated zebrafish R-B neurons with DRG neurons revealed a smaller mean diameter with a larger standard deviation. The distribution of somal diameters for both DRG and R-B neurons appeared unimodal and Gaussian. It should be noted that these data are derived solely from EGFP-expressing neurons. Similar analyses of isolated murine DRGs using phase contrast imaging often showed a multimodal distribution of somal diameters (data not shown). At least two morphologically different populations have been identified in mammalian DRG neurons: those with myelinated axons and large diameter cell body (A-fibers) and those with unmyelinated axons and small diameter somata (C-fibers) [1], [2], [7], [38]. Our attempts to correlate somal diameter with myelination in zebrafish DRG neurons based on immunostaining were unsuccessful perhaps because of different myelination patterns or poorly conserved epitopes between species. Further studies are required to establish how zebrafish DRG neurons relate to their mammalian nociceptive counterparts.

### Voltage-gated Na^+^ channels

The spherical geometry of the isolated DRG neurons was favorable in terms of achieving adequate spatial clamp control. Previous studies of zebrafish *I_Na_* in R-B neurons using nucleated patches [19] or isolated neurons [14] revealed a single rapidly inactivating TTX-sensitive component. In contrast, zebrafish DRG neurons exhibited two kinetically distinct *I_Na_* (rapidly- and slowly-inactivating) TTX-sensitive components. Although the majority of *I_Na_* appeared to decay with a single time constant, the co-existence of multiple Na^+^ channels isoforms in a single neuron cannot be excluded. Interestingly, R-*I_Na_* were present in relatively large, based on membrane capacitance, neurons while S-*I_Na_* was more prevalent in smaller DRG neurons (Fig. 5B). Zebrafish have four primary Na^+^ channel α-subunit isoforms, all of which are duplicated, resulting in eight distinct gene products [39]. RT-PCR results suggest that zebrafish DRG neurons express *zscn1Laa*/*ab*, *zscn8aa/ab*, *zscn4ab* and *zscn5Laa*. The R-*I_Na_* component, based on analogy with R-B cells [19], likely results from *zscn8aa/ab* and/or *zscn1Laa*/*ab* gene products. A possible candidate for the S-*I_Na_* component is *zscn5Laa* based on the slower inactivation kinetics observed when the gene product was heterologously expressed in CHO cells [40]. Although mammalian *SCN5A* is TTX-resistant, both *zscn5Laa/ab* have a tyrosine at the residue cognate to human *SCN5A* cysteine 401 rendering them TTX-sensitive [40]. Further study is required before definitive assignment of this Na^+^ current component can be made.

At least seven distinct Na^+^ channel isoforms are expressed in rodent DRG neurons which contribute to produce two kinetically and pharmacologically distinct *I_Na_* components: one is TTX-sensitive and rapidly inactivates, the second is TTX-resistant with slower inactivation kinetics [41], [42]. Expression of mammalian TTX-resistant Na^+^ channel genes, especially *SCN10A* (Na_V_1.8), occurs primarily in small to medium diameter DRG neurons associated with nociceptive pathways [43]. Mammalian TTX-resistant Na^+^ channels arise from a syntenic gene triplet comprised of *SCN5A*, *SCN10A*, and *SCN11A* that likely resulted from local gene duplication [44]. The latter two genes, coding for Na_V_1.8 and Na_V_1.9, are found almost exclusively in primary sensory neurons. The triplet is also found in birds and lizards but not teleost fish. Thus, *zscn5Laa* may represent the ancestral slowly-inactivating Na^+^ channel of DRG neurons that was latter replaced with *SCN10A* in terrestrial vertebrates following local duplication.

### Voltage-gated Ca^2+^ channels

Zebrafish DRG neuron *I_Ca_* was comprised of relatively few components. Only a few DRG neurons displayed a detectable LVA-*I_Ca_* component and, due to the diminutive amplitude of this component, correlation with somal size was not readily apparent. In a prior study, approximately 30% of zebrafish R-B neurons displayed both LVA- and HVA-*I_Ca_* [14]. Since DRG neurons are thought to functionally replace R-B neuron, a greater percentage of DRG neurons displaying LVA-*I_Ca_* components in juvenile fish was anticipated. It remains possible that LVA-*I_Ca_* components were enriched in the EGFP-negative DRG neurons and thus under represented in the population sampled here. The predominant HVA-*I_Ca_* component was ω-conotoxin GVIA sensitive N-type Ca^2+^ channels, which accounted for about 87% of the total currents amplitude while nifedipine-sensitive L-type Ca^2+^ channels accounted for around 6%. Zebrafish DRG HVA-*I_Ca_* composition and current density were comparable to R-B neurons [14]. A tacit assumption underlying these interpretations is that the toxin/drug pharmacology (i.e., specificity, efficacy and potency) for mammalian and fish Ca^2+^ channel orthologs were similar. In this regard, it should be noted that the L-type Ca^2+^ channel “agonist” FPL64176 produced a prominent inhibition of the zebrafish HVA-*I_Ca_*. Mammalian, in contrast to zebrafish, DRG neurons vary greatly in the expression of LVA- and HVA-*I_Ca_* among small, medium and large diameter cell bodies [6], [45]. T-type *I_Ca_* were observed in small and medium diameter, but not in large diameter DRG cell bodies whereas L-type *I_Ca_* were significantly larger in smaller cell (53%) when compared with medium (6.6%) or large diameter cell bodies (20%). Conversely, N-type *I_Ca_* was similar for all three-size ranges (30, 36 and 25% for small, medium and large cell bodies, respectively).

Zebrafish DRG neuron *I_Ca_* was modulated to some degree by all agonist tested. GABA, OxoM, and DAMGO produced the most consistent and largest amplitude inhibitions; an inhibitory profile different from zebrafish R-B neurons. The cell-to-cell response variability mimics, at least superficially, what is seen in mammalian primary sensory neurons where GPCR expression and ion channel coupling heterogeneity is well established. The two main classifications of HVA-*I_Ca_* inhibition documented in mammalian neurons, voltage-dependent and voltage-independent, were seen in zebrafish neurons. In both zebrafish R-B [14] and DRG neurons (Fig. 10B), the inhibition by DAMGO was voltage-independent as indicated by a decrease in FR during agonist application. As μ-opioid receptor activation in mammalian neurons generally produces voltage-dependent inhibition [46]–[48]. Zebrafish may utilize novel signaling pathways that warrant further investigation.

### Challenges for using isolated zebrafish DRG neurons as a model system

The advantages of using zebrafish as a model system have been mentioned earlier. It is worth re-emphasizing the economy of rearing given the escalating costs of housing higher vertebrates such as mice. There are, however, some challenges (as well as opportunities) that became apparent as our study progressed. First, zebrafish DRG mature slowly with neuronal number increasing, possibly, throughout the life of the organism [10]. This may provide opportunities for investigating mechanisms that provide new neurons into adulthood. However, unlike R-B neurons which occur early in development, body transparency (for imaging) and the applicability of transient gene knockdown techniques (e.g., morpholino antisense oligonucleotides) are sacrificed at later developmental stages. Also, the diminutive size of zebrafish DRG neurons made whole-cell patch-clamp experiments challenging. Second, the duplication of the zebrafish genome makes the study of signaling pathways significantly more complex than in mammals. The signaling pathway for voltage-dependent modulation of N-type Ca^2+^ channels is minimally comprised of a GPCR, heterotrimeric G-protein, and Ca^2+^ channel [49]. Heterotrimeric G-proteins and Ca^2+^ channels are multi-subunit proteins (α, β, and γ for the former, and α_1_, β, and α_2_δ for the latter) with each subunit having multiple isoforms (e.g., >18 G-protein α subunit in mammals) thus providing the basis for many combinatorial possibilities. The zebrafish genome contains duplicates of most subunits thus expanding signaling pathway complexity in a potentially exponential fashion. Moreover, the repertoire of most GPCR classes is greater in zebrafish when compared with *Homo sapiens* [50]. Finally, the sensory modalities conveyed by zebrafish as compared with mammalian DRG neurons is currently unclear. Recently, a group has reported that *erbb3* mutant fish completely lack DRG neurons yet survive to adulthood and display relatively normal swimming and feeding behavior [12], [51]. Thus lateral line neuromasts or cranial trigeminal sensory neurons may compensate for DRG function or, alternatively, play a dominant role in terms of sensory function. The physical environment of aquatic and terrestrial vertebrates differs dramatically which may dictate differences in the sensory modalities transmitted by DRG neurons in each group. Therefore, the role subserved by ion channel modulation in zebrafish sensory physiology and the potential relationship with analogous mammalian systems requires further study.

On the other hand, characteristics of zebrafish DRGs revealed in this study may provide unique opportunities for some types of research. The apparent insignificance of DRG for survival may allow aggressive modulations of DRG functions in behavioral studies. Phylogenetic comparison with other animals may also shed light on the evolutionary adaptation of DRG to environment. Some properties of DRGs, such as those of Na^+^ and Ca^2+^ channels and their modifications by GPCR shown here, are conserved in zebrafish. This suggests that there has been an evolutionary pressure to retain them, and comparison of DRG functions among phylogenetic trees may allow us to link these ion channels to the functions they subserve. Conversely, by focusing on the diversified genes, we may be able to clarify functions of ion channels not appreciated in traditional experimental systems. In particular, environmental factors that led to the expansion of GPCR genes in zebrafish, in DRGs and other neural tissues, will be a highly interesting theme of future research.

## Materials and Methods

### Ethical approval

All of the animal studies and maintenance were approved by the National Institute on Alcohol Abuse and Alcoholism Animal Care and Use Committee.

### Animals

Zebrafish (*Danio rerio*) were housed at 28–29°C in the fish facility on a 14/10 hr light/dark cycle. Embryos were maintained at 28°C in embryo media consisting of 1 mM NaCl and 10^−4^% methylene blue. Staging was based on external morphology [52] and defined as days post-fertilization (dpf). The transgenic fish line, Tg(*Islet2b:EGFP*)^ZC7^, hereafter termed *isl2b:EGFP*, was kindly provided by the late Dr. Chi-Bin Chien (Univ. of Utah Medical Center). Wild-type adult fish (AB or TAB type) were bred with transgenic fish line according to guidelines outlined in the Zebrafish Book (Westerfield, 1995), After crossing, heterozygote embryos were selected using a fluorescence stereomicroscope. Adult male Wister rats (200–220 g, Charles River Laboratories, Inc., MA) were kept in polyacrylic cages and maintained under standard housing condition at room temperature (22–24°C) with 12 h light/dark cycle.

### Preparation of zebrafish DRG neurons

*Isl2b:EGFP* juvenile fish (>50 dpf) were anesthetized in embryo media containing 0.02% tricaine (Sigma-Aldrich, St. Louis, MO). Fish were sacrificed by transection at a caudal level followed by removal of internal organs, skin, and fins. The trunk was transferred to dissociation buffer containing (in mM) 0.6 EDTA, 5.5 d-glucose, 5.4 KCl, 136.8 NaCl, 5.5 NaHCO_3_, and 10 mg/ml collagenase CLS4 (Worthington Biochemical, Lakewood, NJ) and incubated for 30 min at room temperature. After incubation, partially dissociated muscle tissues were triturated with a Pasteur pipette until both spinal cord and notochord were exposed. Before proceeding, verification of EGFP-labeled DRG pairs in each trunk segment was accomplished using a fluorescence microscope. This partially prepared central trunk region was transferred to dissociation buffer containing 2 mg/ml trypsin TRL (Worthington Biochemical, Lakewood, NJ) and incubated for 20 min at room temperature (21–24°C). After the incubation, DRG neurons were carefully dissociated with a smaller bore Pasteur pipette in culture medium consisting of 60% L-15 media (Invitrogen, Carlsbad, CA), 1 mM Na-HEPES (Sigma-Aldrich), 1% penicillin-streptomycin (Invitrogen), and 0.5% horse serum (Invitrogen). Dissociated DRG neurons were plated on poly-l-lysine (Sigma-Aldrich) coated tissue culture dishes and maintained overnight at room temperature prior to recording.

### Preparation of Superior Cervical Ganglion (SCG) neurons

Single SCG neurons were enzymatically dissociated as described previously [53]. Briefly, rats were anesthetized by CO_2_ inhalation and subsequently decapitated prior to dissection. The SCG were removed bilaterally, cut into small pieces, and incubated in the modified Earles' balanced salt solution (EBSS) containing 2 mg/ml collagenase CLS4 (Worthington Biochemical, Lakewood, NJ), 0.7 mg/ml trypsin TRL (Worthington Biochemical) and 0.05 mg/ml DNase I (Sigma–Aldrich, St. Louis, MO) at 37°C for 60 min. The EBSS was modified by adding 3.6 g/L glucose and 10 mM HEPES. After incubation, neurons were dissociated by vigorous shaking of the flask. The dissociated cells were washed twice, transferred to minimum essential medium (MEM) containing 10% fetal bovine serum and 1% penicillin-streptomycin (all from Invitrogen), plated on poly-l-lysine (Sigma–Aldrich) coated tissue culture dishes and maintained in a humidified 95% air/5% CO_2_ incubator at 37°C.

### Electrophysiological recording

Both voltage-gated Na^+^ and Ca^2+^ currents (*I_Na_* and *I_Ca_*, respectively) were recorded using the conventional whole-cell patch-clamp configuration [54]. Patch electrodes were fabricated from borosilicate glass capillaries (1.5 mm outer diameter, 0.84 mm inner diameter; WPI Inc. Sarasota, FL) using a model P-97 micropipette puller (Sutter Instrument Company, Novato, CA). The patch electrodes were coated with Sylgard® 184 (Dow Corning, Midland, MI) and fire-polished to a final resistance of ∼8–10 MΩ when filled with the pipette solution described below. After rupturing the cell membrane, the mean access resistance was 19.9±0.5 MΩ. The cell membrane capacitance was cancelled and series resistance was routinely compensated (>85% for both prediction and compensation; lag set to 10 µs) with an Axopatch 200B patch-clamp amplifier (Molecular Devices, Sunnyvale, CA). The bath was grounded by an Ag/AgCl pellet connected via a 0.15 M NaCl/agar bridge. Voltage protocol generation and data acquisition were performed using custom-designed software (S5) on a Macintosh G4 computer (Apple Computer Inc., Cupertino, CA) equipped with an ITC-18 data acquisition interface (InstruTECH Corporation, Bellmore, NY). Currents were filtered at 2 kHz (−3 dB) using a four-pole low-pass Bessel filter, digitized at 10 kHz with a 16-bit analog-to digital converter in the ITC-18 data acquisition interface, and stored on the computer. All drugs and bath solution were applied to single neurons via a gravity-fed fused silica capillary tube connected to an array of seven polyethylene tubes. The bath solution was applied from this system between drug applications to compensate for flow-induced artifacts. All recordings were performed at room temperature (21–24°C).

### Solutions and chemicals

For recording of *I_Na_*, the external solution contained (mM) 127 NaCl, 3 KCl, 20 TEA-Cl, 5 MnCl_2_, and 5 HEPES (pH 7.4, 300 mOsm/kg H_2_O). The pipette solution contained (mM) 120 *N*-methyl-d-glucamine, 20 TEA-OH, 11 EGTA, 1 CaCl_2_, 10 HEPES, 10 glucose, 4 Na_2_ATP, 0.3 Na_2_GTP, and 5 Tris-creatine phosphate (pH 7.2, 290 mOsm/kg H_2_O). To isolate *I_Ca_*, patch electrodes were filled with a solution containing (in mM) 120 N-methyl-d-glucamine, 20 tetraethylammonium hydroxide (TEA-OH), 11 EGTA, 10 HEPES, 1 CaCl_2_, 20 HCl, 4 MgATP, 0.1 Na_2_GTP and 14 Tris-creatine phosphate (pH 7.2, 295 mOsm/kg H_2_O). The bath solution contained (in mM) 140 methanesulfonic acid, 145 TEA-OH, 10 HEPES, 10 glucose, 10 CaCl_2_, and 0.0003 tetrodotoxin (pH 7.4, 320 mOsm/kg H_2_O). Stock solutions were made for the following drugs: γ-aminobutyric acid (GABA), l-glutamic acid hydrochloride, oxotremorine M, FPL64176 (all from TOCRIS Cookson Ltd., Ellisville, MO), nifedipine (EMD chemicals, Gibbstown, NJ), tetrodotoxin (TTX), ω-agatoxin IVA, ω-conotoxin GVIA, SNX-482 (all from Alomone Labs, Jerusalem, Israel), (±)-norepinephrine, serotonin hydrochloride (5-hydroxytryptamine, 5-HT) and CdCl_2_ (all from Sigma-Aldrich). Drugs were prepared in distilled water, except for nifedipine and FPL64176, which were dissolved in dimethyl sulfoxide (DMSO). All drugs were diluted to the final concentrations from stock solutions on the day of the experiment.

### Immunohistochemistry

Juvenile *isl2b:EGFP* fish were anesthetized with tricaine and head, fins, organs and skin removed. Samples were fixed overnight (4°C) with 4% paraformaldehyde in phosphate buffered saline (PBS) solution and then incubated in 30% sucrose overnight at 4°C. Samples were snap frozen by embedding with Tissue Tek OCT compound (Sakura Finetech, Torrance, CA) and stored at −80°C. Frozen blocks were sectioned transversely (15 µm) on a cryostat (CM3050; Leica, Nussloch, Germany), mounted on SuperFrost Plus slides (VWR international, Radnor, PA), and air-dried. After washing with PBS, sections were permeabilized by incubation in PBS containing 0.4% Triton X-100 (PBST) for 30 min and blocked with the addition of 4% bovine serum albumin (BSA) for 4 hours. Sections were incubated in primary antibodies diluted in blocking solution overnight at 4°C. Primary antibodies used were anti-HuC/HuD neuronal protein (1∶500; mouse antibody, Invitrogen, Cat.# A21271), anti-GABAb R1 (1∶500; goat antibody, Santa Cruz, Cat.# SC-14006), anti-5-HT (1∶500; rabbit, Immunostar, Cat.# 20080). Sections were washed five times (1 hr each) in PBST and incubated overnight with Alexa Fluor® 568 conjugated secondary antibodies (Invitrogen, Cat.# A21069) at 4°C. After five times washes in PBST, sections were observed with the confocal microscope. Alexa Fluor® 568 conjugated Isolectin GS-IB_4_ (1∶400, Invitrogen, Cat.# 121412) was used to identify neuronal subpopulations in zebrafish DRG sections.

### Imaging

Brightfield or fluorescent images of zebrafish were acquired on the Olympus MVX fluorescent stereomicroscope with a CCD camera (Olympus DP70). Confocal images were done using a confocal microscope (Zeiss LSM510 META, Thornwood, NY) with ×10 (0.3 N.A.) or ×25 (0.8 N.A.) water immersion objective. For EGFP fluorescence, excitation was 488 nm and emission was bandpass-filtered between 500–550 nm and Alexa Fluor® 568 was imaged using 565 nm excitation and emission was bandpass-filtered between 576–704 nm. For double imaging of Alexa Fluor® 568 and GFP, sequential scanning was performed. Dissociated cells were imaged on an inverted fluorescence microscope (Eclipse TE2000-U, Nikon, Melville, NY). All images were imported into ImageJ (http://imagej.nih.gov/ij, USA) for analysis and contrast adjustment.

### RT-PCR analysis

A dissociated DRG cluster was collected by suction into a fire-polished glass capillary filled with diethylpyrocarbonate (DEPC)-treated water. The pipette tip was then broken inside a PCR tube containing components for reverse transcription. Total RNA was reverse-transcribed using a QuantiTect Reverse Transcription kit (Qiagen, Valencia, CA). A 5 µl aliquot of cDNA product (total 20 µl) was transferred to reaction tubes for amplification of β-actin or EGFP and the remaining product (10 µl) was used for *zscn* transcripts amplification using 2× GoTag Hot Start Polymerase Green Master Mix (Promega, Madison, WI). PCR was performed for 40 cycles consisting of 94°C for 30 s, 59°C for 30 s, and 72°C for 90 s. The final extension was performed at 72°C for 5 min. Forward and reverse primer sequences were targeted to different exons allowing discrimination of products amplified from mRNA versus genomic DNA. The following primer pairs were used: *zscn1Laa* (Gene bank Accession # NM_200132, 659 bp, for 5′-cccgcgagtcccttaaggcc-3′, rev 5′-ggttttcagccctgggacgact-3′): *zscn1Lab* (Accession # NM_001044895, 380 bp, for 5′-ggcccaaaggcgagagacgg-3′, rev 5′-cgtgtattccacgttcttggccca-3′): *zscn4aa* (Accession # NM_001039825, 573 bp for 5′-accagtgtgttccgccgctt-3′, rev 5′-tgtgacgtacgccatgctgatcac-3′): *zscn4ab* (Accession # NM_001045065, 605 bp, for 5′-tgctccctcccacaggcacc-3′, rev 5′-gaggtacgccatgctgatgacca-3′): *zscn8aa* (Accession # NM_131628, 964 bp, for 5′-cggtgctacctattggccgac-3′, rev 5′-gccaacaatggtcttcagacctgga-3′): *zscn8ab* (Accession # NM_001045183, 707 bp, for 5′-aggccccgacagcttcaagag-3′, rev 5′-ctcccacaatggttttcagccgtgg-3′): *zscn5Laa* (Accession # NM_001044922, 479 bp, for 5′-cccggaaacgggagcttcgtc-3′, rev 5′-aaagccctctgctccttgcgca-3′): *zscn5Lab* (Accession # NM_001045123, 461 bp, for 5′-gtgacgctgggaaatgtccgga-3′, rev 5′-tgacagcggccgggagcttt-3′): β-actin (Accession # AF025305, 1001 bp, for 5′-ctccattgttggacgacccag-3′, rev 5′-cagactcatcgtactcctgc-3′): EGFP (345 bp, for 5′-cctacggcgtgcagtgcttcagc-3′, rev 5′-cggcgagctgcacgctgccgtcctc-3′). Specificity of primers was tested with total RNA prepared from whole zebrafish brain or muscles. The PCR products were analyzed by agarose gel electrophoresis and visualized using SYBR® safe DNA gel stain (Invitrogen).

### Data analysis

Current traces were analyzed using Igor Pro version 6.04 (WaveMetrics, Portland, OR) and statistical tests were performed with GraphPad Prism 5 for Mac OS X (GraphPad Software, La Jolla, CA) or Igor Pro. The percentage inhibition of *I_Ca_* (%) was determined using the equation (*I_con_*−*I_drug_*)/*I_con_*×100, where *I_con_* and *I_drug_* were the *I_Ca_* amplitudes before and after drug application, respectively. Concentration-response curve were constructed by fitting experimental data to the Hill equation: *B* = *B*_max_/(1+(*IC_50_*/[drug])*^n^*), where *B* is the fraction blocked, *B*_max_ is the maximal block, *IC_50_* is the half-maximal inhibitory concentration of the drug applied, and *n* is the Hill slope. Statistical significance between two groups was evaluated using either unpaired t-test with Welch's correction for unequal variance or the Mann-Whitney *U*-test as noted. *P*<0.05 was considered significant. The modified Wald method (http://www.graphpad.com/quickcalcs/ConfInterval1.cfm) was used to compute confidence intervals of a proportion.
